# Genomic selection with fixed-effect markers improves the prediction accuracy for Capsaicinoid contents in *Capsicum annuum*

**DOI:** 10.1093/hr/uhac204

**Published:** 2022-09-13

**Authors:** Geon Woo Kim, Ju-Pyo Hong, Hea-Young Lee, Jin-Kyung Kwon, Dong-Am Kim, Byoung-Cheorl Kang

**Affiliations:** Department of Agriculture, Forestry and Bioresources, Research Institute of Agriculture and Life Sciences, Plant Genomics Breeding Institute, College of Agriculture and Life Sciences, Seoul National University, Seoul 08826, Republic of Korea; Department of Agriculture, Forestry and Bioresources, Research Institute of Agriculture and Life Sciences, Plant Genomics Breeding Institute, College of Agriculture and Life Sciences, Seoul National University, Seoul 08826, Republic of Korea; Department of Agriculture, Forestry and Bioresources, Research Institute of Agriculture and Life Sciences, Plant Genomics Breeding Institute, College of Agriculture and Life Sciences, Seoul National University, Seoul 08826, Republic of Korea; Department of Agriculture, Forestry and Bioresources, Research Institute of Agriculture and Life Sciences, Plant Genomics Breeding Institute, College of Agriculture and Life Sciences, Seoul National University, Seoul 08826, Republic of Korea; R&D Center, Hana Seed Co., Ltd., Anseong 17601, Republic of Korea; Department of Agriculture, Forestry and Bioresources, Research Institute of Agriculture and Life Sciences, Plant Genomics Breeding Institute, College of Agriculture and Life Sciences, Seoul National University, Seoul 08826, Republic of Korea

## Abstract

Capsaicinoids provide chili peppers (*Capsicum* spp.) with their characteristic pungency. Several structural and transcription factor genes are known to control capsaicinoid contents in pepper. However, many other genes also regulating capsaicinoid contents remain unknown, making it difficult to develop pepper cultivars with different levels of capsaicinoids. Genomic selection (GS) uses genome-wide random markers (including many in undiscovered genes) for a trait to improve selection efficiency. In this study, we predicted the capsaicinoid contents of pepper breeding lines using several GS models trained with genotypic and phenotypic data from a training population. We used a core collection of 351 *Capsicum* accessions and 96 breeding lines as training and testing populations, respectively. To obtain the optimal number of single nucleotide polymorphism (SNP) markers for GS, we tested various numbers of genome-wide SNP markers based on linkage disequilibrium. We obtained the highest mean prediction accuracy (0.550) for different models using 3294 SNP markers. Using this marker set, we conducted GWAS and selected 25 markers that were associated with capsaicinoid biosynthesis genes and quantitative trait loci for capsaicinoid contents. Finally, to develop more accurate prediction models, we obtained SNP markers from GWAS as fixed-effect markers for GS, where 3294 genome-wide SNPs were employed. When four to five fixed-effect markers from GWAS were used as fixed effects, the RKHS and RR-BLUP models showed accuracies of 0.696 and 0.689, respectively. Our results lay the foundation for developing pepper cultivars with various capsaicinoid levels using GS for capsaicinoid contents.

## Introduction

Chili peppers (*Capsicum* spp.) are used as spices due to their pungent characteristics. In 2020, the worldwide production of fresh and dry peppers was approximately 38 million tons and 4.2 million tons, respectively (FAOSTAT). The spicy characteristics of peppers are derived from capsaicinoids, which have analgesic [1], anticarcinogenic [2], and antiobesity effects [3].

Capsaicinoids include capsaicin and dihydrocapsaicin, which is a saturated hydrocarbon type of capsaicin. Various structural genes controlling capsaicinoid production have been discovered, including *putative AMINOTRANSFERASE* (*p-AMT*) [4], *Pungency 1* (*Pun1*) [5], and *Ketoacyl-ACP reductase* (*KR*) [6]. Various MYB domain transcription factor genes and other unknown genes also control capsaicinoid contents [7–9]. Capsaicinoid contents are also affected by light conditions, nutrient availability, biotic stress, and various environmental conditions [10–12].

The presence/absence of pungency can easily be determined using molecular markers derived from known genes such as *p-AMT* and *Pun1*. However, no reliable molecular markers are currently available for predicting capsaicinoid contents, since various genetic and environmental factors contribute to capsaicinoids production in pepper fruits. To identify genetic factors associated with these traits, quantitative trait locus (QTL) mapping was conducted using bi-parental and genome-wide association study (GWAS) populations [13]. However, the ability to detect minor QTLs is limited because of a large number of minor QTLs with small effects. Therefore, a complementary method that considers both major and minor genetic factors should be employed [14].

Genomic selection (GS) was initially developed to improve quantitative traits and to overcome the limitations of QTL mapping in animals [15]. GS uses various models trained using training populations with available phenotypic and genotypic data. The trained models calculate the genomic estimated breeding values (GEBVs) of traits in breeding populations using only genotypic data. GS was first applied to predict the quality of Canadian Holstein cows and to select good individuals [16]. Subsequently, GS has been successfully applied to other animals and to plants [17, 18].

Ridge-regression best linear unbiased prediction (RR-BLUP) and Bayesian methods are the most commonly used GS models. These models include a penalization step to prevent all markers from having the same effect during the model training phase [15]. However, markers associated with major QTLs sometimes have low marker effects because these effects are randomly adjusted without considering the effects of the markers that contribute to the traits. Therefore, the trained model does not always predict the target phenotype accurately. To circumvent this problem and to develop more accurate models, simulations can be conducted to validate the model’s performance by designating the important genes as fixed effects [19]. When a few genes influenced a trait by 10% or more, the model was more accurate when these genes were designated as fixed effects. Later research on rice (*Oryza sativa*) and winter wheat (*Triticum aestivum*) showed that the prediction accuracy of horticultural traits increased when previously discovered trait-related genes or markers derived from GWAS were assigned as fixed effects [20, 21].

The only GS study on peppers performed to date predicted fruit-related traits [22]. This study used many models to identify various parameters that influence prediction accuracy, including the population structure of the training and testing populations, the types of models, and the number of markers [22]. However, to date, no study has been performed to improve the performance of models in horticultural crops using fixed effects. Therefore, in the current study, we combined various models and genotypic data to construct optimal prediction models of capsaicinoid contents using commercial elite pepper lines. We then applied the GWAS results to GS and designated significant SNPs as fixed effects to improve model performance. We evaluated the performance of different models using various marker sets, demonstrating the power of using GS with fixed effects in actual breeding.

## Results

### Calculating representative phenotypes and adjusting the number of markers

**Figure 1:**
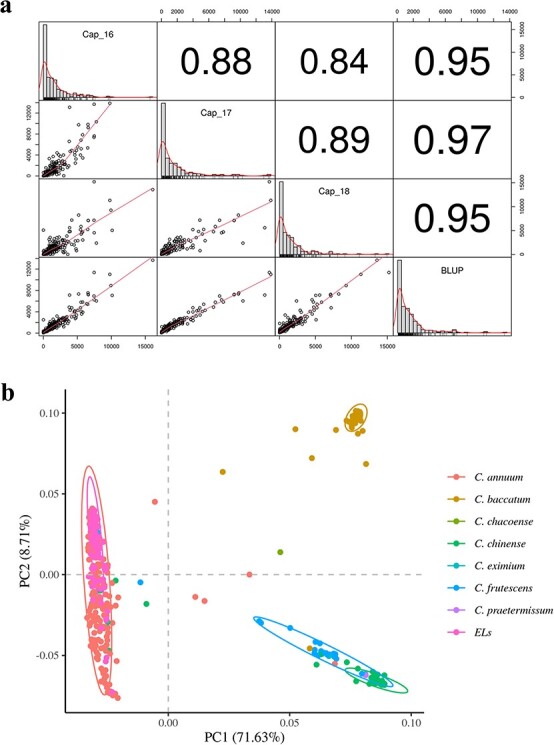
Test for the phenotypic representativeness of BLUP and PCA using the core collection and elite lines (ELs). a BLUP was calculated based on years and growth environments. These values were highly correlated with every year, indicating that they are representative values. b The first principle component explained 71.6% of the variance across the populations. The populations were divided by species based on both components. The ELs were grouped together with *Capsicum annuum* in the core collections. The analysis was conducted using the R package ‘rrblup’[32].

We calculated the best linear unbiased prediction (BLUP) values of capsaicinoid contents in each accession and used these values to minimize the variation due to different biological repeats, growth environments, and years of study. The correlations across each year were > 0.84, but the correlations between BLUP values and each year were 0.95 or higher (Fig. 1a). This observation suggests that the BLUP values are representative for each year.

We combined raw reads from genotyping-by-sequencing and targeted sequencing into a single variant call format (vcf) file and filtered the data. All plant materials used in this study thus included the same set of markers. We adjusted the number of markers based on the degree of linkage disequilibrium (LD) to produce six sets of markers, ranging from 896 to 18 029 markers per set.

### Population structure analysis

We performed a principal component analysis (PCA) of the core collection and elite lines (Fig. 1b). We obtained a good separation between accessions with the top two principal components. The first major component accounted for 71.6% of the standing population variance, while the second component accounted for another 8.7%. The five main clusters are shown in circles in Fig. 1b. These clusters largely grouped the accessions according to their species. Indeed, we identified the elite lines used as the testing set as the *C. annuum* cluster. *C. frutescens* and *C. chinense* grouped closely together, and *Capsicum baccatum* was separated from the other clusters. Therefore, the *C. annuum* complex containing *C. annuum*, *C. chinense*, and *C. frutescens* was used as a material for subsequent analyses.

### Genomic prediction of capsaicinoid contents

**Figure 2:**
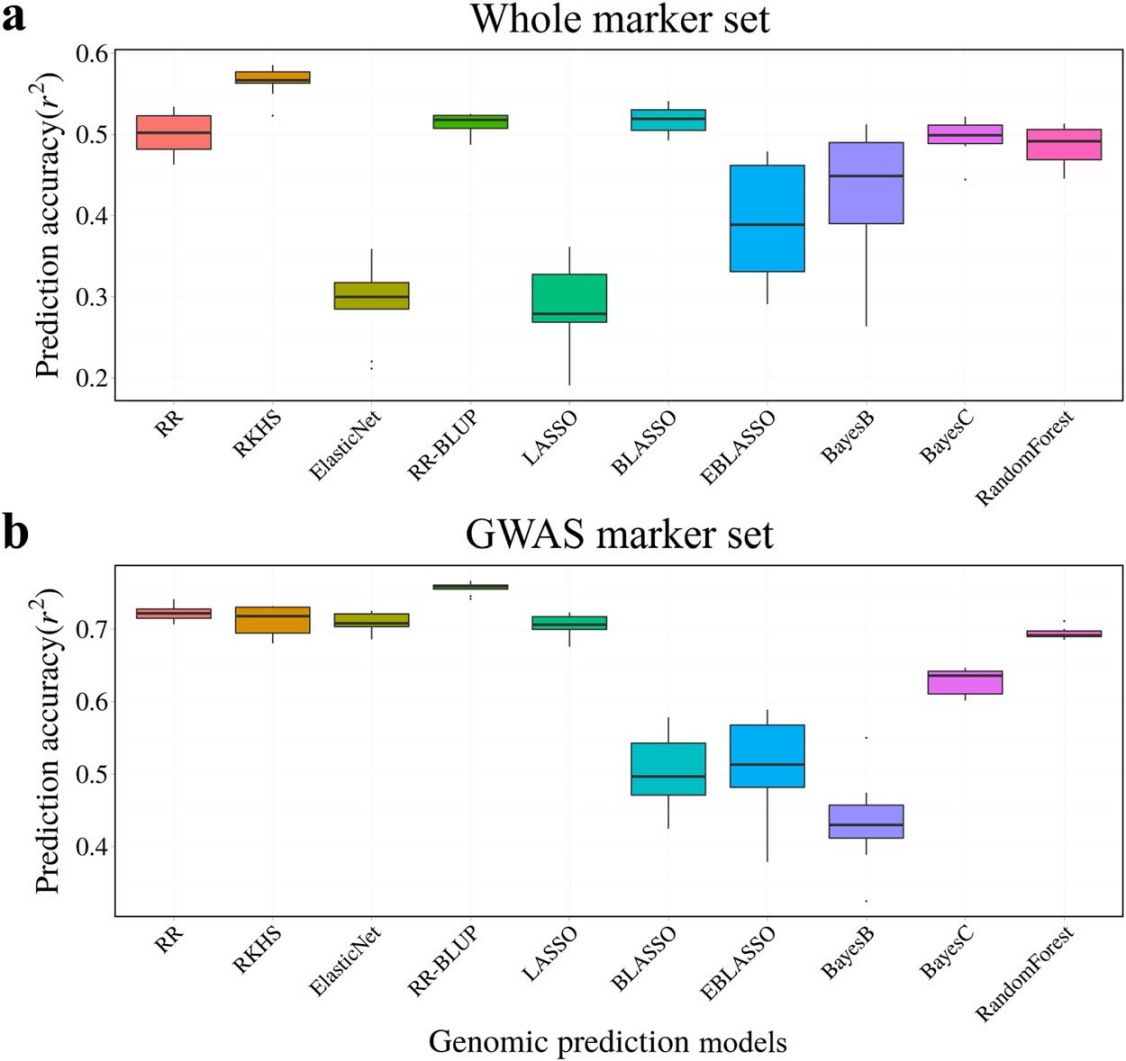
Validation of ten different models by the 10-fold cross-validation method in the core collection. a 10-Fold cross-validation was conducted to validate the performance of each model for predicting capsaicinoid contents in the *C. annuum* complex of the core collection before using these models to predict the phenotypes of the test population. b 10-Fold cross-validation was performed to verify that the 98-marker set could be applied to other test populations outside of the elite lines. RR, ridge regression; RKHS, reproducing kernel Hilbert space; LASSO, the least absolute shrinkage and selection operator; BLASSO, Bayesian LASSO; EBLASSO, extended BLASSO.

We validated ten GS models that were trained with all markers by 10-fold cross-validation to determine their performance before predicting the capsaicinoid contents of the elite lines (Fig. 2a). The prediction accuracy of 10-fold cross-validation was highly dependent on the model employed. We obtained the highest mean prediction accuracy of 0.565 for the RKHS model, with the LASSO model returning the lowest accuracy of 0.283, with an overall mean model accuracy of 0.448, indicating that most models perform fairly well.

For GS, we trained ten models using the six marker sets with different numbers of SNP markers and predicted the capsaicinoid contents using only elite lines. The accuracy of each model varied depending on the type of model and the number of markers. When trained with all markers, the EN model showed the highest accuracy (0.539). The accuracy of the RKHS, RR-BLUP, BayesB, and BayesC models increased as the number of markers decreased, with the lowest values obtained using all markers (18029) and the highest values obtained when the models were trained with 3294 markers (RKHS, 0.661; RR-BLUP, 0.658; BayesB, 0.657; and BayesC, 0.660). The accuracies decreased when fewer than 3294 markers were used. The RR and EBLASSO models trained with 7496 markers showed the highest prediction accuracies (0.653 and 0.480, respectively). We observed the highest prediction accuracies for the BLASSO and RF models when they were trained with 11 796 and 5207 markers, respectively. The prediction accuracy of LASSO decreased as the number of SNPs decreased, down to 5207 markers. However, the prediction accuracy peaked (0.400) when using 3294 markers. Among all prediction results, the RF model trained with 5207 markers showed the highest prediction accuracy (0.665; Table 1).

The RR-BLUP models demonstrated the best mean accuracy and the lowest standard deviations across all marker sets. However, the LASSO and BayesB models exhibited the lowest mean accuracy (0.308) and the largest standard deviations (0.181; Table S1). Across all marker sets, models trained with 3249 markers had high accuracies and low standard deviations (Table S2). To save the cost of genotyping in GS, it is necessary to optimize the number of markers for genotyping while maintaining enough prediction accuracy. Thus, we selected the 3249 markers for subsequent study.

### Genomic selection using markers derived from GWAS for capsaicinoid content

To obtain markers from GWAS for use in GS, we conducted GWAS using 18 029 markers and seven GWAS models for capsaicinoid contents. The number of SNP markers associated with capsaicinoid contents varied depending on the GWAS model: six SNPs from BLINK; ten from FarmCPU and MLMM; 91 from ECMLM and MLM; 63 from CMLM; 119 from GLM. After removing the redundant markers, we retained 146 nonredundant SNPs (Fig. S1). To remove false positive markers, we conducted ANOVA using the phenotype and genotype of each SNP marker, yielding 98 markers with *p*-values <0.05 (Table S3). Among these 98 significant markers, 26 markers were associated with QTL blocks or located near previously reported capsaicinoid biosynthesis genes [13, 23–25]. These 26 markers were distributed on chromosomes 1, 2, 3, 4, 5, 6, 7, and 10 and were associated with ten QTL blocks and 18 genes. A total of nine genes were identified on chromosomes 2, 4, 6, and 7, each overlapping with one QTL block. Chromosome 5 also had only a QTL block containing a significant marker. And the *putative pyruvate dehydrogenase E3 subunit* (*EU616580.1*) gene was located around this marker. Chromosome 1 included three QTL blocks and two genes, while chromosome 3 contained two QTLs and five genes. We detected no QTL block on chromosome 10, but did detect one gene associated with a significant marker on this chromosome.

When using these 98 significant GWAS markers as the training data for GS, the average accuracy of the models was 0.583, representing the highest mean accuracy, with a standard deviation of 0.044. Even though we used fewer than 100 markers, the models performed better when trained with 98 GWAS markers compared to 18 029 genome-wide markers. Furthermore, the performance of GS using these markers did not fluctuate across different models (Table S4).

We performed ten-fold cross-validation to determine whether the results showing good predictive accuracy with a small number of GWAS markers were limited to the current study. Ten-fold cross-validation using 98 markers showed that most models had high accuracies and that the RR-BLUP model showed the best performance (Fig. 2b). Based on this analysis, these GWAS markers could be used to accurately predict phenotypes in populations other than those analyzed in the current study.

**Table 1:** Prediction accuracies of the models trained with different numbers of genome-wide markers in the elite lines

LD (*r^2^*)	Num. of SNPs	Model									
		RR	RKHS	EN	RR-BLUP	LASSO	BLASSO	EBLASSO	BayesB	BayesC	RF
1	18 029	0.539	0.577	0.539	0.609	0.349	0.571	0.451	0.356	0.581	0.651
0.9	11 796	0.625	0.6	0.335	0.631	0.311	0.63	0.373	0.621	0.622	0.625
0.7	7496	0.653	0.613	0.315	0.639	0.321	0.563	0.48	0.637	0.64	0.636
0.5	5207	0.6	0.652	0.388	0.638	0.267	0.613	0.404	0.632	0.636	0.665
0.3	3294	0.563	0.661	0.425	0.658	0.4	0.559	0.356	0.657	0.66	0.563
0.1	896	0.426	0.56	0.226	0.551	0.198	0.47	0.265	0.18	0.507	0.426

### Genomic selection with fixed effects

To validate the performance of the fixed effects in GS, we used the RKHS and RR-BLUP models. The genotypic data set used for model training was the 3387-marker set, which was composed of 3294 genome-wide markers (LD 0.3) and 98 significant GWAS markers (five markers were common).

We performed GWAS to identify fixed-effect markers and selected 88 significant markers (Fig. S2 and Table S6). When we compared these markers to the 98 GWAS markers described above, we identified 84 common markers. When we compared QTL positions and candidate genes for the 88 markers, 25 markers corresponded to 26 candidate genes, as shown in Table 2.

**Table 2:** The physical positions of markers associated with QTLs or genes controlling capsaicinoid contents, and the prediction accuracies for the fixed-effect markers

Name	Chr.	Start	End	Annotation	Reference
*PD-dicap1.3*	1	231 321 032	254 240 579	.	[13]
*AF127796.1*	1	234 216 596	234 219 084	*acyl carrier protein (Acl1)*	Gene
5_93211388	1	239 582 729	.	.	GWAS
*TH-cap1.5*	1	283 296 805	320 053 625	.	[13]
5_29079727	1	303 714 390	.	.	GWAS
*EU616569.1*	1	304 631 073	304 641 350	*putative 3-oxoacyl-(acyl-carrier-protein) synthase III*	Gene
*TH-cap2.2*	2	126 513 242	167 435 903	.	[13]
14_49166074	2	127 312 721	.	.	GWAS
14_29575792	2	146 903 003	.	.	GWAS
*AY819029.1*	2	155 365 412	155 367 392	*acyltransferase (Pun1) gene, complete cds*	Gene
14_19305298	2	157 173 497	.	.	GWAS
*pAMT* (*CA03g08530*)	3	37 228 188	37 232 589	*putative aminotransferase*	Gene
7_38620302	3	38 298 393	.	.	GWAS
*PD-cap3*	3	208 538 594	271 096 557	.	[13]
7_230338271	3	230 016 385	.	.	GWAS
7_235620528	3	235 298 642	.	.	GWAS
*EU616574.1*	3	249 051 706	249 056 357	*putative NADH-dependent glutamate synthase 1*	Gene
7_266174959	3	265 853 073	.	.	GWAS
*TH-total3.3*	3	272 436 493	281 912 421	.	[13]
7_275650607	3	275 328 721	.	.	GWAS
*EU616555.1*	3	280 471 817	280 474 536	*putative cinnamoyl-CoA reductase*	Gene
*EU616565.1*	3	281 457 373	281 462 454	*putative hydroxycinnamoyl transferase*	Gene
*EU616540.1*	3	281 797 865	281 802 323	*putative 4-coumarate-CoA ligase 2*	Gene
*4CL* (*CA03g30500*)	3	281 798 105	281 802 230	*4-coumarate-CoA ligase*	Gene
*PD-total4.2*	4	32 243 580	185 523 937	.	[13]
1_154297181	4	94 096 053	.	.	GWAS
1_135298897	4	113 094 337	.	.	GWAS
1_97746893	4	150 646 341	.	.	GWAS
*EU616579.1*	4	164 461 292	164 466 996	*putative pyruvate dehydrogenase E2 subunit*	Gene
1_83051978	4	164 733 172	.	.	GWAS
1_78145242	4	169 639 908	.	.	GWAS
1_77539838	4	170 245 312	.	.	GWAS
*EU616547.1*	4	179 661 422	179 663 383	*putative acetolactate synthase*	Gene
*EU616557.1*	4	182 391 838	182 393 687	*putative cytochrome reductase*	Gene
1_63078891	4	184 706 259	.	.	GWAS
*Yarnes_5*	5	212 947 567	235 981 655	.	[24]
12_235645145	5	235 980 559	.	.	GWAS
*EU616580.1*	5	236 739 999	236 744 613	*putative pyruvate dehydrogenase E3 subunit*	Gene
*Lee_6*	6	150 249 645	188 229 109	.	[45]
6_154345277	6	154 345 376	.	.	GWAS
*EU616548.1*	6	188 222 723	188 224 992	*putative branched-chain alpha-keto acid dehydrogenase E1 alpha subunit*	Gene
*Ben_7_1*	7	102 332 599	193 879 410	.	[23]
4_142537535	7	121 027 103	.	.	GWAS
4_129542534	7	134 022 104	.	.	GWAS
4_127051517	7	136 513 121	.	.	GWAS
4_127051516	7	136 513 122	.	.	GWAS
*EU616577.1*	7	168 829 974	168 832 740	*putative pyruvate dehydrogenase E1 alpha subunit*	Gene
*EU616572.1*	7	190 031 185	190 039 562	*putative long-chain acyl-CoA synthetase*	Gene
*EU616581.1*	10	219 504 152	219 505 324	*putative S-adenosylmethionine synthetase*	Gene
3_220454148	10	220 454 049	.	.	GWAS
3_232610354	10	232 135 281	.	.	GWAS
*LC379875.1*	10	232 726 829	232 731 286	*ketoacyl-ACP reductase*	Gene

**Figure 3:**
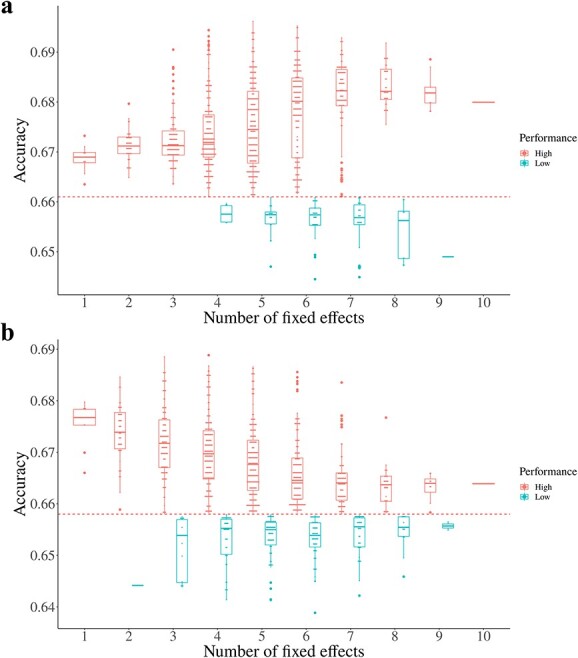
Prediction accuracies of the models using different combinations of fixed-effect markers in the elite lines. a Accuracy of the RKHS model based on the fixed effect combination of the top ten markers among QTLs and gene-related markers. b Accuracy of the RR-BLUP model based on the fixed effect combination of the top ten markers among QTLs and gene-related markers. The x-axis shows the number of fixed-effect markers, and the y-axis shows the prediction accuracy. The red dashed line shows the prediction accuracy of the models without fixed effects. The red dots represent cases where the prediction accuracy is higher than the accuracy of the model without the fixed effect, while the blue dots represent cases where the accuracy is less than the accuracy of the model without the fixed effect.

We selected ten markers for fixed effects from these 25 markers based on their ranking, as described in the Methods. We calculated the fixed effect of each marker by comparing the accuracy of the models with or without the fixed-effect markers (Table 3). We also tested every possible combination of ten selected markers, from two to nine random markers. The prediction accuracies varied depending on the number and type of marker combination. The average accuracy of the RKHS model increased from 0.669 to 0.680 as the number of fixed-effect markers increased up to five. Finally, when five specific markers (14_29575792, 14_49166074, 7_230338271, 1_154297181, and 3_220454148) were employed as fixed effects, this model displayed the highest accuracy (0.696) across all fixed-effect combinations (Fig. 3a). Compared to the result without fixed effects, this resulted in an accuracy gain of 5.3%. The average of the top ten best RKHS models with fixed effects was 0.694, and each combination commonly contained markers 14_29575792 and 14_49166074.

**Table 3:** Information about the fixed-effect markers, including performance

Name	Chr.	Pos.	Ref.	Alt.	RKHS	RR-BLUP
5_93211388	1	239 582 729	T	A	0.659	0.680
5_29079727	1	303 714 390	T	C	0.652	0.675
14_49166074	2	127 312 721	T	C	0.673	0.592
14_29575792	2	146 903 003	C	T	0.664	0.666
14_19305298	2	157 173 497	G	C	0.652	0.675
7_38620302	3	38 298 393	A	T	0.605	0.603
7_230338271	3	230 016 385	G	A	0.670	0.677
7_235620528	3	235 298 642	C	T	0.666	0.540
7_266174959	3	265 853 073	C	T	0.659	0.675
7_275650607	3	275 328 721	T	C	0.670	0.532
1_154297181	4	94 096 053	T	C	0.671	0.566
1_135298897	4	113 094 337	C	T	0.659	0.675
1_97746893	4	150 646 341	T	C	0.652	0.675
1_83051978	4	164 733 172	G	A	0.668	0.563
1_78145242	4	169 639 908	A	G	0.661	0.676
1_77539838	4	170 245 312	A	T	0.660	0.678
1_63078891	4	184 706 259	C	T	0.661	0.670
12_235645145	5	235 980 559	A	G	0.657	0.678
6_154345277	6	154 345 376	T	G	0.652	0.679
4_142537535	7	121 027 103	G	A	0.622	0.632
4_129542534	7	134 022 104	G	A	0.659	0.613
4_127051517	7	136 513 121	A	G	0.668	0.530
4_127051516	7	136 513 122	G	A	0.668	0.529
3_220454148	10	220 454 049	G	A	0.670	0.547
3_232610354	10	232 135 281	A	G	0.652	0.675

Ref, reference allele; Alt, alternative allele.

The average accuracy of the RR-BLUP model decreased as the number of fixed-effect markers increased, with the highest accuracy observed when using four fixed-effect markers. When four markers (5_93211388, 14_29575792, 7_230338271, and 6_154345277) were designated as fixed effects, the maximum accuracy was 0.689 (Fig. 3b), representing a 4.7% improvement in the accuracy of the RR-BLUP model over that without the fixed effect. The average of the top ten best RR-BLUP models with fixed effects was 0.687, and each combination included markers 14_29575792 and 5_93211388 (Table 6).

## Discussion

In this study, we conducted GS for capsaicinoid contents in pepper using genome-wide markers together with markers derivedfrom GWAS. A previous study showed that the prediction accuracies of GS vary depending on the parameters of the GS models [22]. In the current study, we demonstrated the possibility of using GWAS results for GS to improve the prediction accuracy. When we conducted GS without fixed effects, GS with 3294 markers showed high prediction accuracies regardless of the model (Table 1). When marker sets obtained from GWAS were used in GS, the GS models were as accurate as models using genome-wide markers, even though we used fewer than 100 markers (Table S4). To increase the performance of GS, we conducted GS using markers associated with QTLs or genes that are known to influence capsaicinoid biosynthesis as fixed effects (Table 2). Although the ability of the fixed effects to improve model accuracy varied based on the type of model and the combination of fixed-effect markers, adding fixed effects to the model was an effective way to increase the prediction accuracy (Fig. 3).

The first GS study published in peppers also used the *C. annuum* complex as a training set [22]. The *C. annuum* complex is considered to be suitable as a training set for GS because it is genetically similar to breeding populations. In previous studies, *C. annuum*, *C. chinense*, and *C. frutescens* were classified as the *C. annuum* complex, as they show similar morphological traits. These species cluster in the *C. annuum* complex because they evolved from a common ancestor [26, 27]. Although these species are different, they show similar morphological traits, reflecting their past and present selection based on common traits [27].

Significant SNPs obtained from GWAS varied depending on the model (Fig. S1 and Fig. S2). A previous study using “GAPIT3” package demonstrated that the statistical power of the models was highest for BLINK, followed (in decreasing order) by FarmCPU, MLMM, ECMLM, CMLM, MLM, and GLM (Table S5) [28]. MLM is a form of GLM in which kinship information is added as random co-factors [29]. ECMLM increases the statistical power by finding the best combination between various kinship algorithms and subgroups of kinship data generated by CMLM [30]. Even though ECMLM finds the optimal kinship data, the results were the same as those of MLM. This finding implies that the kinship data used in MLM is the best kinship data of the core collection. The significant SNPs in the ANOVA were discovered in the BLINK, FarmCPU, MLMM, and ECMLM models, which accounted for the most significant SNPs discovered among the models (Table S3 and Table S6), suggesting that various markers are linked to traits. Furthermore, these models have strong statistical power. As a consequence, the combined results of the multiple GWAS models will provide additional opportunities to investigate trait-related mutations.

In this study, ten different models were used and compared to select the models to check the performance of models with the fixed-effect (Table S7). A previous study showed that there were various interactions among genes or QTLs in different environments through a genome-wide by environment interaction study model [31]. Thus, the RKHS model performed best in 10-fold cross-validation (Fig. 2a) because capsaicinoid contents are influenced by many genes, and RKHS can detect epistasis between genes [32]. RR, RR-BLUP, BLASO, BayesC, and RF also showed excellent performance. When making predictions using the same marker sets used for cross-validation, RF showed the highest accuracy because RF model also could consider the epistasis (0.651; Table 1). In addition to RF, models that performed well in cross-validation also performed well in trait prediction in the testing population. RF model was the ensemble model which merge the results of decision trees. These trees found the interaction between the genetic variance and the combinations of markers. This feature resulted in the ability to consider the epistasis [33]. By contrast, EN, LASSO, EBLAOS, and BayesB, which performed poorly in cross-validation, also performed poorly in prediction, except for EN in the testing population. When many markers were used, EN performed worse on cross-validation but better on prediction than LASSO because EN is designed to overcome the instability of LASSO by using many markers [34–36]; these characteristics of EN were shown in this study as like the previous study while making predictions in the testing population. EN performed well when the prediction was performed using a large number of markers. However, when the number of markers decreased, EN’s performance was similar to that of LASSO. In the Bayesian models, BayesB and BayesC models showed the higher prediction accuracy than those predicted by BLASSO and EBLASSO models (Table 1). BayesB and BayesC models used the scaled inverse chi-square distribution as prior distribution to estimate the marker effect, beside BLASSO and EBLASSO used the Laplace prior distribution (Table S7) [15, 37–39]. Based on these results, it could be inferred that the marker effect variance was closer to the scaled inverse chi-square distribution.

Numerous genome-wide markers were required for GS in previous studies [22, 40]. However, in the current study, the GS models using markers obtained from GWAS performed as well as those using genome-wide markers. We first performed 10-fold cross-validation using marker sets obtained from GWAS. The cross-validation results showed that RR-BLUP models had the highest accuracy and BayesB model had the lowest accuracy (Fig. 2b). RR-BLUP model, which calculated all marker effects to predict phenotype, could calculate the marker effects well because 96 markers derived from GWAS had a small number of markers and phenotypic information. On the other hand, BayesB model selected the markers to use for prediction and ignored the others. Because of this characteristic of BayesB model, many markers with phenotypic information were ignored and it caused decreasing accuracy (Table S7). These results indicate that GS using marker sets obtained from GWAS will result in high prediction accuracies for capsaicinoid contents in other testing populations. We hypothesize that the GWAS marker sets performed well because the SNPs were selected based on their associations with the trait of interest. These results are consistent with those of previous studies in which the model accuracy improved when a trait-related marker was added or when the model was trained only using trait-related markers [41, 42]. Therefore, even if most genome-wide markers are excluded from the modeling process, markers selected by GWAS could be sufficient to predict capsaicinoid contents. However, the GWAS marker set should be tested in various testing populations to confirm the results.

When we compared the RR-BLUP and RKHS results, both models exhibited high accuracy until the number of markers decreased to 896. However, when the number of markers was less than 896 with an LD value of 0.10, both models showed the lowest accuracy (Table 1). A previous study showed that self-pollinated crops are strongly affected by additive × additive epistasis and that cross-fertilized crops are more strongly affected by dominant gene action [43]. Since pepper is a self-pollinated crop, the core collection and elite lines used in this study are genetically homozygous. Therefore, the traits are more strongly affected by epistasis between markers (not dominant gene actions) in these populations. RKHS was more accurate than RR-BLUP only when we used a small number of markers for modeling, perhaps due to overfitting when calculating epistasis as the number of markers increases.

We selected 25 candidate fixed-effect markers that were associated with previously reported QTLs or capsaicinoid biosynthesis genes (Table 2). Among these markers, we used all possible combinations of the top ten markers as fixed effects in each model. The prediction accuracy varied depending on the combinations of fixed effects based on which or how many markers were selected. We achieved the highest accuracy using the RKHS model when assigning five markers as fixed effects; we observed the highest accuracy using the RR-BLUP model when four markers were assigned as fixed effects (Fig. 3). These results are in line with the recommendation that ten or fewer major genes should be designated as fixed effects [19].

The markers that were commonly used in the fixed-effect combinations with good results are located within the same QTL block as the capsaicinoid biosynthesis genes (Table S8). Marker 14_29575792, which enhanced the accuracies of both models, is located in QTL block TH-cap2.2. This block also includes the *Pun1* gene (AY819029.1), encoding an enzyme that condenses two precursors, vanillylamine and 8-methyl-6-nonenoyl-CoA, during capsaicinoid biosynthesis. Marker 14_49166074, which improved the RKHS model, is located in the same QTL block as *Pun1*. Finally, marker 5_93211388, which improved the RR-BLUP model, is located in the same QTL block (PD-dicap1.3) as the *acyl carrier protein* gene (AF127796.1), whose encoded protein acts as a cofactor in fatty acid biosynthesis to synthesize the fatty acid chains of capsaicinoids [44]. These results are consistent with the finding that using combinations of genetic regions rather than designating a single genetic region as a fixed effect improved the accuracy of a model used to predict the heading date and plant height of wheat [20].

In conclusion, we conducted GS using various models to predict capsaicinoid contents. Although the marker set derived from GWAS was small, most models displayed relatively high prediction accuracies for capsaicinoid contents when these markers were used for GS. Furthermore, markers associated with the target trait increased the prediction accuracies by up to 10% for the RR-BLUP and RKHS models when these markers were included as fixed effects. The method developed in this study should facilitate the breeding of *Capsicum* cultivars with various capsaicinoid contents.

## Materials and methods

### Plant materials

The core pepper (*Capsicum* spp.) collection from the Horticultural Crops Breeding and Genetics Laboratory at Seoul National University, Republic of Korea [45] was used in this study. The core collection comprises 351 accessions, including 224 *C. annuum*, 49 *C. chinense*, 24 *C. frutescens*, 50 *C. baccatum*, two *C. chacoense*, one *Calliostoma eximium*, and one *C. praetermissum* accessions. The core collection was grown at the Rural Development Administration (Jeonju, Korea) in 2015 and 2017 and at Hana Seed Corporation (Anseong, Korea) in 2018 [22]. Three plants per accession were sown in early March and transplanted in May to grow in the greenhouse. Most fruits were harvested in August, but some samples were harvested earlier or later, depending on maturity. All 96 elite lines from Hana Seed Corporation were *C. annuum* except for two hybrids between *C. annuum* and *C. baccatum*.

### Genotyping methods

The genotypic data for the core collection were variant calling data obtained in previous studies [22, 45]. The core collection was genotyped by sequencing as previously described [45]. Commercial elite lines were genotyped by targeted sequencing. A library of elite lines was constructed using an Allegro Targeted Genotyping Kit (Tecan Genomics Inc., Redwood City, CA, USA). The genomic DNA fragments were digested by a restriction enzyme mix and ligated to library adaptors following user guide of Allegro Targeted Genotyping (version 3). The fragments were amplified using selective primers. The newly constructed libraries were pooled into three tubes and sequenced in separate lanes on a NextSeq500 system (Illumina, San Diego, CA, USA) at DNA Link (Seoul, Republic of Korea). Variant calling, trimming, and filtering were conducted to obtain genotypic data as previously described [22]. The raw data from both populations were trimmed and filtered by quality based on a minimum read length (80 bp) and minimum quality score (Q20) using CLC Genomics Workbench v6.5 (Qiagen, Aarhus, Denmark). The resulting clean reads of both populations were aligned to the *C. annuum* “Dempsey” genome (unpublished) using Burrows-Wheeler Aligner (BWA) and sorted and grouped using Genome Analysis Toolkit (GATK). Variant calling was conducted on the sorted alignment data using GATK Haplotype Caller 3.8, and filtering was conducted using GATK VariantFIltration (MQ < 40.0, SOR > 3.000, QD < 2.00, FS > 60.000, MQRankSum< −12.500, ReadPosRankSum< −8.000). SNPs with a missing ratio > 70% and a minor allele frequency (MAF) of 0.05 of were removed from the filtered VCF data using VCFtools. These genotypic data were imputed by BEAGLE using the R package “synbreed” [46]. The imputed genotypic data from the core collection and elite lines included 18 029 SNPs. This SNP data set was separated into six sub-genotypic datasets with different numbers of SNPs based on linkage disequilibrium (LD).

### Ligh-performance liquid chromatography (HPLC) analysis of capsaicinoid contents

The capsaicinoid contents of the core collection were measured using HPLC for three years: 2015, 2017, and 2018. The capsaicinoid contents of the commercial elite lines were measured in 2018. Capsaicinoids were extracted for HPLC analysis using an ethyl acetate and acetone mixture [47]. In detail, more than three fruits were harvested for each repeat. After freeze drying, 1.5 mL of a mixture of ethyl acetate and acetone (6:4, v/v) was poured onto 0.1 g of finely ground tissue, and the mixture was incubated at 37°C in a shaking incubator at 200 rpm for 24 h. The incubated mixture was dried using an automatic environmental speedvac system (AES1010; Operon, Gimpo, Korea). The pellet was resuspended in 1 mL of 99.9% pure methanol (Honeywell - Burdick & Jackson, Muskegon, MI, USA). After filtering through a 0.2-μm syringe filter (Macherey-Nagel Inc., Bethlehem, PA, USA), HPLC was conducted at NICEM (Seoul, Korea). The best linear unbiased prediction (BLUP) values were calculated for representative values of each accession in the core collection based on the year, location, and number of iterations using the R package “lme4” [48]. The capsaicin and dihydrocapsaicin contents of the elite lines were represented by the average values of replicates, and the total capsaicinoid contents of each line were calculated based on the sum of capsaicin and dihydrocapsaicin contents.

### Analysis of population structure

Principal component analysis (PCA) was performed to determine the relationship between the core collections to be used as a training set and the elite lines to be used as a testing set. Based on the availability of genotypic data, only one accession and one elite line were not selected among a core collection of 351 accessions and 96 elite lines. A matrix of the relationships between the 445 samples was calculated by the “A.mat” function in the R package “rrBLUP” [32]. The influence of each component was analyzed based on the relationship matrix using the “prcomp” function in the R package “stats”. Each individual was represented as a dot in dotplots using the R package “ggplot2” for each calculated deviation.

### Genomic selection

The various models were trained with a training population consisting of 284 accessions of the *C. annuum* complex (*C. annuum*, *C. chinense*, and *C. frutescens*) that was genetically related to the testing population based on a different number of markers. The phenotypic data were the BLUP values of capsaicinoid contents. Ten prediction models were used: ridge-regression best linear unbiased prediction (RR-BLUP), reproducing kernel Hilbert space (RKHS), ridge regression (RR), least absolute shrinkage and selection operator (LASSO), elastic net (EN), Bayesian LASSO (BLASSO), extended Bayesian LASSO (EBLASSO), BayesB, BayesC, and random forest (RF) [15, 22, 32, 35, 37–39, 49, 50].

After model training, 10-fold cross-validation was performed to select the best GS models. Ten-fold cross-validation was conducted as follows: 1) divide the training set into ten subsets, 2) train with nine subsets and estimate the remaining subset, and 3) repeat ten times by changing the composition of the nine subsets used for training.

The capsaicinoid contents of the elite lines were predicted by the trained models based on the genotypic data of the elite lines. The marker list of the elite line and core collection was the same. The prediction accuracies between the predicted capsaicinoid contents and the actual measurements were estimated by Pearson’s correlation analysis.

### GS using markers selected from GWAS

To discover the variants associated with capsaicinoid contents, GWAS was employed to select markers that were related to capsaicinoid contents. GWAS was conducted via seven different models using the R package “GAPIT 3” [28] with 284 accessions in the *C. annuum* complex used as the training population. The phenotypic data were the BLUP values of capsaicinoids for each accession, and the genotypic data were the total marker set (18 029 markers). These variants were used to validate how accurate models trained by only a marker set related to the target trait are. Then, to select the fixed-effect markers, GWAS was conducted using the marker set which contained the LD 0.3 markers (3298 markers) and 98 significant markers in the *C. annuum* complex to avoid the false negatives derived from population structure.

The seven GWAS models were Bayesian-information and Linkage-disequilibrium Iteratively Nested Keyway (BLINK), Fixed and random model Circulating Probability Unification (FarmCPU), multiple loci mixed model (MLMM), Mixed linear model (MLM), Compressed MLM (CMLM), Enriched CMLM (ECMLM), and General linear model (GLM). The threshold was calculated by dividing the number of SNPs by 0.05 according to the Bonferroni correction method. The significant markers derived from each GWAS model were combined to a marker set to complement the false negatives depending on the models and to improve the possibility of finding the variants highly associated with the capsaicinoid biosynthesis.

To construct a non-redundant marker set from combined marker set described in above paragraph, duplicate markers were removed for markers that exceeded the Bonferroni correction calculated from each model. Markers in a non-redundant marker set were evaluated by analysis of variance (ANOVA) to test significant differences in the mean capsaicinoid contents of each accession. ANOVA was performed using the “aov” function in the R package “stats”. SNPs whose *p*-values were < 0.05 were selected as significant SNPs.

The GS models were trained with SNP markers selected from GWAS for capsaicinoid contents in the core collection as described above. To verify that this marker set could be applied to other breeding populations, 10-fold cross-validation was conducted, and the capsaicinoid contents of the elite lines were predicted using these trained models.

To select the fixed-effect markers from significant GWAS markers, the performance of each marker was tested by adding GWAS markers one by one to each model. The ranking of the fixed-effect markers was determined based on the accuracies of the models. The top-ten best markers were then selected, and combinations of these selected markers were tested as fixed effects in each model.

To validate the performance of models with fixed-effect markers, the models were trained in the core collection with the combination of selected fixed-effect markers and the phenotypes of the elite lines were predicted using the “kinship.BLUP” function of the R package “rrBLUP”. The accuracy of the models was determined by calculating the correlation between the predicted and actual values in the elite lines using the “cor” function of the R package “stats”.

## Acknowledgements

This work was carried out with the support of the “Cooperative Research Program for Agriculture Science and Technology Development (Project No. PJ015881)” Rural Development Administration, Republic of Korea.

## Author Contributions

G.K. conducted all analyses and wrote the paper. J.H. performed phenotyping and genotyping of plant populations. H.L. and D.K. designed the plant population. J.K. supervised the genomic predictions. B.K. supervised the overall study and revised the manuscript. All authors revised and approved the final version of the paper.

## Data Availability

All data, which were used in this study, were included in these article and supplementary data.

## Conflict of Interest

The authors declare that the research was conducted in the absence of any commercial or financial relationships that could be construed as a potential conflict of interest.

## Supplementary data

Supplementary data is available at *Horticulture Research* online.

## Supplementary Material

supp_data_uhac204
