# Supplementary material for: Genomic selection with fixed-effect markers improves the prediction accuracy for Capsaicinoid contents in Capsicum annuum
Source: Hortic Res. 2022 Sep 13;9:uhac204. doi: 10.1093/hr/uhac204 (PMC9714256; doi:10.1093/hr/uhac204)
Supplement: supp_data_uhac204 [file supp_data_uhac204.zip › Horticulture research_GS_TableS5.docx]

**Table S5. Seven GWAS models were used in this study.**

| Method | Testing marker | Number of steps | Model | Kinship |
| --- | --- | --- | --- | --- |
| GLM | Single locus | One | Fixed | NA |
| MLM | Single locus | One | Mixed | All markers |
| CMLM | Single locus | One | Mixed | Individuals clustered into groups |
| ECMLM | Single locus | One | Mixed | Individuals clustered into groups by enrichment |
| MLMM | Multiple loci | Iterative | Mixed | All markers |
| FarmCPU | Multiple loci | Iterative | Fixed and Mixed | Pseudo QTNs |
| BLINK | Multiple loci | Iterative | Fixed | NA |

The above table was modified from the table described in Wang and Zhang, 2021^1^.

Seven GWAS models used in this study were Blink, FarmCPU, MLMM, ECMLM, CMLM, MLM, and GLM, and the statistical power of the models is decreased in the order in which they are written above^1^. GLM directly detects the relationship between phenotype and genotype. This model uses the population structure as a cofactor^2^. GLM detects the largest number of SNPs, as proposed by the model but with the lowest statistical power. SNPs detected in this model contain a significant number of false positives. MLM is similar to GLM in that kinship information is added as random co-factors^3^. CMLM creates subgroups based on kinship data. CMLM simplifies kinship data using new kinship data between subgroups^4^. ECMLM finds the best combination between various kinship algorithms and those that generate subgroups in CMLM^5^. Statistical power is further increased by finding the best combination of algorithms. MLMM is suitable for application when multiple genetic factors regulate traits. The GWAS results will have more false positives if multiple genetic factors control traits. One way to reduce the false positives is to increase the number of cofactors. Thus, iterations of adding cofactors and performing GWAS reduce false positives^6^. The FarmCPU detects the relationship between bins and phenotypes after configuring the ambient markers into one bin. Configuring markers into one bin reduces false positives and the number of markers, reducing the problem of model overfitting^7^. BLINK is a model that improves the FarmCPU model to increase statistical accuracy and computational efficiency. By converting nearby markers into one bin like FarmCPU, markers independent of target traits are sometimes included in the bins. This indicates the bin is associated with the trait, however, the exact location cannot be specified. Therefore, it detects the relationship between each marker and its traits. Moreover, BLINK improves computational efficiency by reconstructing a set of the most trait-associated markers according to LDs^8^.

References

1 Wang J, Zhang Z. GAPIT Version 3: Boosting Power and Accuracy for Genomic Association and Prediction. *Genomics, Proteomics & Bioinformatics* 2021; **19**: 629–640.

2 Purcell S, Neale B, Todd-Brown K *et al.* PLINK: A tool set for whole-genome association and population-based linkage analyses. *American Journal of Human Genetics* 2007; **81**: 559–575.

3 Yu J, Pressoir G, Briggs WH *et al.* A unified mixed-model method for association mapping that accounts for multiple levels of relatedness. *Nature Genetics* 2006; **38**: 203–208.

4 Zhang Z, Ersoz E, Lai C-Q *et al.* Mixed linear model approach adapted for genome-wide association studies. *Nature Genetics* 2010; **42**: 355–360.

5 Li M, Liu X, Bradbury P *et al.* Enrichment of statistical power for genome-wide association studies. *BMC Biology* 2014; **12**: 73.

6 Segura V, Vilhjálmsson BJ, Platt A *et al.* An efficient multi-locus mixed-model approach for genome-wide association studies in structured populations. *Nat Genet* 2012; **44**: 825.

7 Liu X, Huang M, Fan B, Buckler ES, Zhang Z. Iterative Usage of Fixed and Random Effect Models for Powerful and Efficient Genome-Wide Association Studies. *PLOS Genetics* 2016; **12**: e1005767.

8 Huang M, Liu X, Zhou Y, Summers RM, Zhang Z. BLINK: a package for the next level of genome-wide association studies with both individuals and markers in the millions. *Gigascience* 2019; **8**. doi:10.1093/gigascience/giy154.
