# Supplementary material for: Genomic selection with fixed-effect markers improves the prediction accuracy for Capsaicinoid contents in Capsicum annuum
Source: Hortic Res. 2022 Sep 13;9:uhac204. doi: 10.1093/hr/uhac204 (PMC9714256; doi:10.1093/hr/uhac204)
Supplement: supp_data_uhac204 [file supp_data_uhac204.zip › Horticulture research_GS_TableS7.docx]

**Table S7. Classification of models used for genomic selection.**

| Category |  |  | Model | Reference |
| --- | --- | --- | --- | --- |
| Linear type  (Parametric) | Shrinkage model | Penalty method | Ridge regression (RR) | Friedman et al., 2010^1^ |
|  |  |  | Elastic net (EN) |  |
|  |  | Bayesian method | Bayesian ridge regression (RR-BLUP) | Endelman, 2011^2^ |
|  | Variable selection model | Penalty method | Least absolute shrinkage and selection operator (LASSO) | Friedman et al., 2010^1^ |
|  |  | Bayesian method | BayesB | Meuwissen et al., 2001^3^ |
|  |  |  | BayesC | Habier et al., 2011^4^ |
|  |  |  | Bayesian LASSO (BLASSO) | Park and Casella, 2008^5^ |
|  |  |  | Extended BLASSO (EBLASSO) | Mutshinda and Sillanpaa, 2010^6^ |
| Non-linear type  (Non-parametric) | Kernel model |  | Reproducing kernel Hilbert space (RKHS) | Endelman, 2011^2^ |
|  | Machine learning |  | Random forest (RF) | Breiman, 2001^7^ |

In animal breeding, the traditional best linear unbiased prediction (BLUP) model predicted the phenotype based on the kinship matrix. It is effective in predicting offspring phenotypes using pedigree information. However, the prediction accuracy approached zero as generation advances. It implied that prediction should be applied only if the genotype of the training population is similar to the genotype of the breeding population. Compared with BLUP, RR-BLUP predicts phenotype based on genomic relationship, so it guarantees a certain level of phenotype prediction accuracy even if the difference between training and breeding population existed^8^.

Reproducing kernel Hilbert space (RKHS) model uses the kernel to consider the epistatic effects. The kernel was used because the interaction among genetic factors was too complex to make models for these directly. Thus, the RKHS model is the method that considers the marker interaction by estimating marker effects through the kernel and could detect epistasis^9^.

Penalty methods decrease the variance of estimates and increased computational efficiency by shrinking the marker effects to a data set with much more markers than individuals. The Ridge regression (RR) model, one of the penalty methods, also shrinks the marker effect but not shrink to zero. The least absolute shrinkage and selection operator (LASSO) model ignores most marker effects by shrinking marker effects to zero and uses the rest of the marker effects. This characteristic could enhance the prediction accuracy but sometimes misses the important marker highly associated with target traits. The elastic net (EN) model was developed to complement this problem. EN model combines the RR and LASSO models and works like the LASSO model, but with less sparsity^1,10^.

Bayesian methods designate the prior distribution for marker effects to detect markers effectively associated with the target trait. In a previous study, these methods showed higher prediction accuracy by selecting the prior distribution, although the realistic distribution of marker effects differed from the prior distribution^3^. BayesB model uses the scaled inverse chi-square distribution as prior distribution^3^. However, the scale parameter mainly affects the shrinkage because the prior distribution of the BayesB model is determined by a few degrees of freedom and scale parameters. To solve this problem by reducing the influence of the scale parameter, the BayesC model uses the scaled inverse chi-square prior distribution with 4.2 degrees of freedom as a common single effect variance instead of locus-specific variance^4^. Bayesian LASSO (BLASSO) uses independent Laplace distribution as prior distribution and shows the middle characteristic of RR and LASSO models^5^. Because only one parameter of BLASSO controls the model sparsity, which adjusts how many marker effects are set to zero, and parameter shrinkage, which determines how much marker effects are reduced, however, the adaptiveness of the model is deficient. Therefore, the extended BLASSO (EBLASSO) model complements the problem of the BLASSO model. The EBLASSO model separated the model sparsity and parameter shrinkage each and improved the adaptiveness and predictive performance^6^.

Random forest (RF) is the ensemble model that combines many decision tree results. To make the decision trees, some markers are selected randomly from whole markers and genetic variance is determined by combinations of the selected markers. This process is iterated making many decision trees. This model could consider the epistasis effect among markers and reduce the predictive error because the genetic variance derived from various marker combinations are combined^11^.

Reference

1 Friedman J, Hastie T, Tibshirani R. Regularization Paths for Generalized Linear Models via Coordinate Descent. *Journal of Statistical Software* 2010; **33**: 1–22.

2 Endelman JB. Ridge Regression and Other Kernels for Genomic Selection with R Package rrBLUP. *The Plant Genome* 2011; **4**: 250–255.

3 Meuwissen THE, Hayes BJ, Goddard ME. Prediction of Total Genetic Value Using Genome-Wide Dense Marker Maps. *Genetics* 2001; **157**: 1819–1829.

4 Habier D, Fernando RL, Kizilkaya K, Garrick DJ. Extension of the bayesian alphabet for genomic selection. *BMC Bioinformatics* 2011; **12**: 186.

5 Park T, Casella G. The Bayesian Lasso. *J Am Stat Assoc* 2008; **103**: 681–686.

6 Mutshinda CM, Sillanpää MJ. Extended Bayesian LASSO for Multiple Quantitative Trait Loci Mapping and Unobserved Phenotype Prediction. *Genetics* 2010; **186**: 1067–1075.

7 Breiman L. Random Forests. *Machine Learning* 2001; **45**: 5–32.

8 Clark SA, Hickey JM, Daetwyler HD, van der Werf JHJ. The importance of information on relatives for the prediction of genomic breeding values and the implications for the makeup of reference data sets in livestock breeding schemes. *Genet Sel Evol* 2012; **44**. doi:10.1186/1297-9686-44-4.

9 González-Recio O, Gianola D, Long N, Weigel KA, Rosa GJM, Avendaño S. Nonparametric methods for incorporating genomic information into genetic evaluations: An application to mortality in broilers. *Genetics* 2008; **178**. doi:10.1534/genetics.107.084293.

10 James, G., Witten, D., Hastie, T., Tibshirani R. *An Introduction to Statistical Learning - with Applications in R | Gareth James | Springer*. 2013.

11 Montesinos López OA, Montesinos López A, Crossa J. Random Forest for Genomic Prediction. In: *Multivariate Statistical Machine Learning Methods for Genomic Prediction*. Springer International Publishing: Cham, 2022, pp 633–681.
