# Supplementary figures and images for: Genomic selection with fixed-effect markers improves the prediction accuracy for Capsaicinoid contents in Capsicum annuum
Source: Hortic Res. 2022 Sep 13;9:uhac204. doi: 10.1093/hr/uhac204 (PMC9714256; doi:10.1093/hr/uhac204)

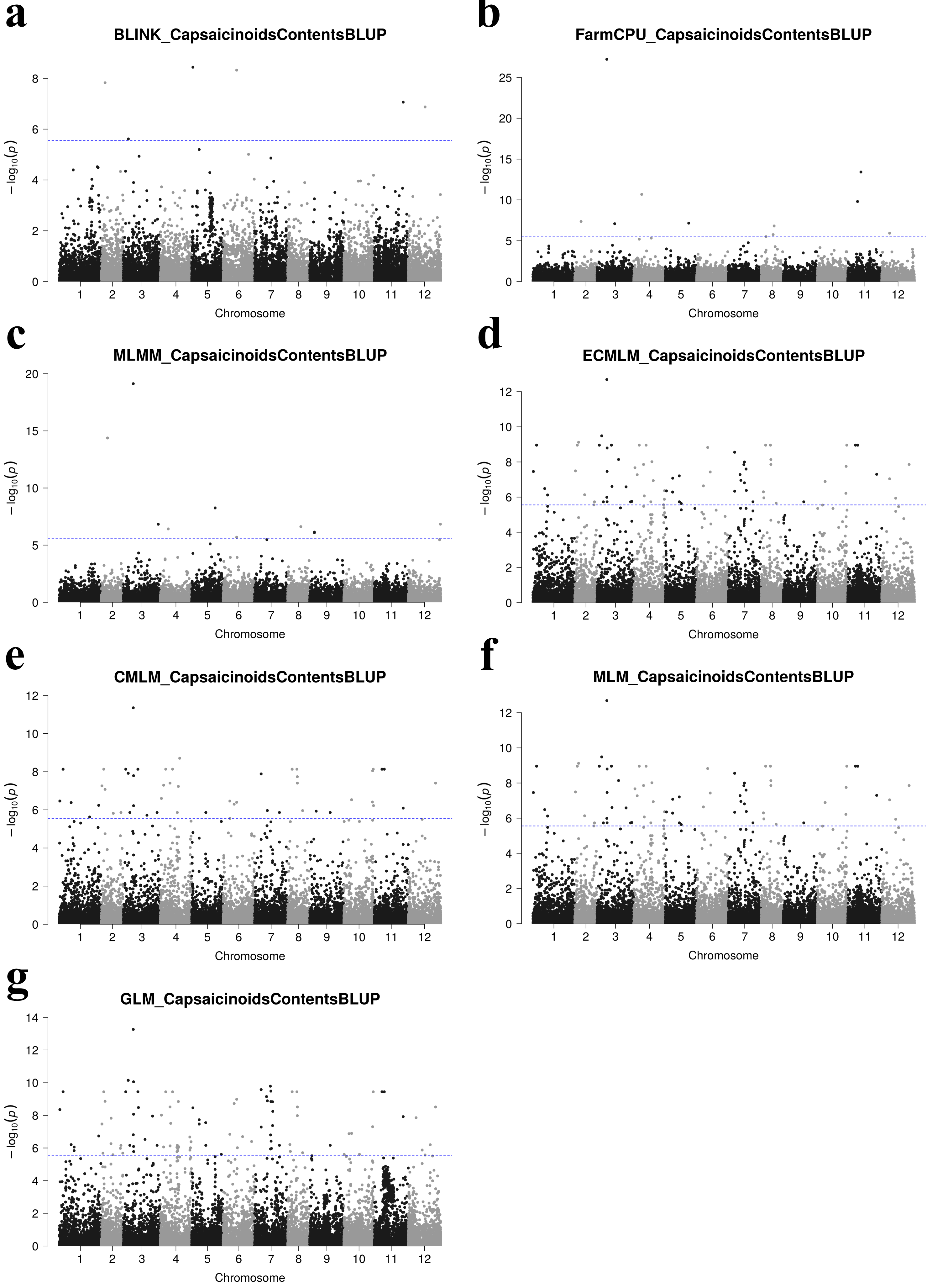

Supplement: supp_data_uhac204 [file supp_data_uhac204.zip › 20220503_Fig. S1_GWAS_whole marker set_capsaicinoid BLUP.png]

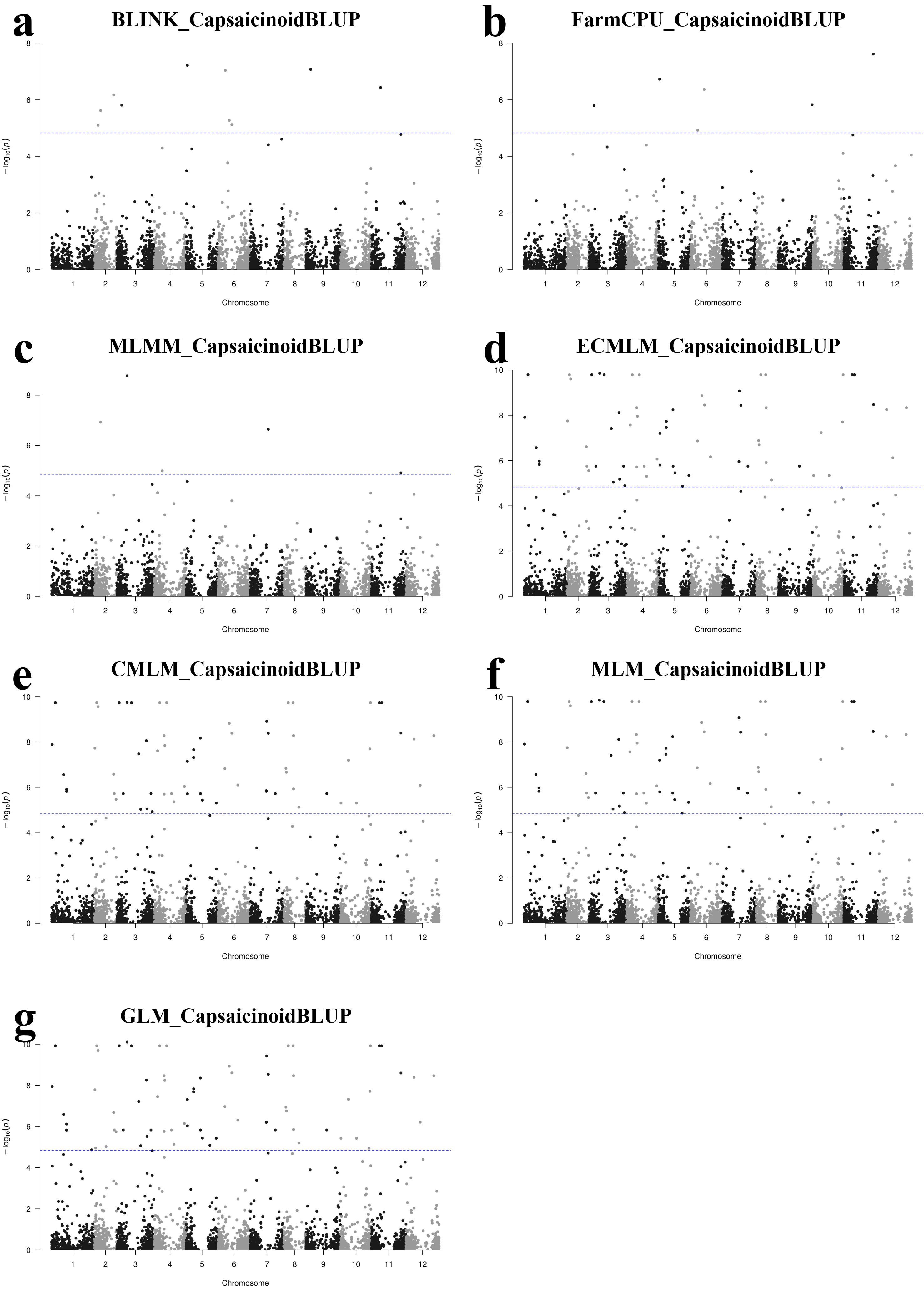

Supplement: supp_data_uhac204 [file supp_data_uhac204.zip › 20220503_Fig. S2_GWAS_3387 marker set.png]
